# A novel technique to determine the cell type specific response within an *in vitro* co-culture model *via* multi-colour flow cytometry

**DOI:** 10.1038/s41598-017-00369-4

**Published:** 2017-03-27

**Authors:** Martin J. D. Clift, Kleanthis Fytianos, Dimitri Vanhecke, Sandra Hočevar, Alke Petri-Fink, Barbara Rothen-Rutishauser

**Affiliations:** 1 BioNanomaterials, Adolphe Merkle Institute, University of Fribourg, Fribourg, Switzerland; 2 In Vitro Toxicology Group, Swansea University Medical School, Wales, UK; 3 Department of Chemistry, University of Fribourg, Fribourg, Switzerland

## Abstract

Determination of the cell type specific response is essential towards understanding the cellular mechanisms associated with disease states as well as assessing cell-based targeting of effective therapeutic agents. Recently, there have been increased calls for advanced *in vitro* multi-cellular models that provide reliable and valuable tools correlative to *in vivo*. In this pursuit the ability to assess the cell type specific response is imperative. Herein, we report a novel approach towards resolving each specific cell type of a multi-cellular model representing the human lung epithelial tissue barrier *via* multi-colour flow cytometry (FACS). We proved *via* ≤ five-colour FACS that the manipulation of this *in vitro* model allowed each cell type to be resolved with no impact upon cell viability. Subsequently, four-colour FACS verified the ability to determine the biochemical effect (*e.g.* oxidative stress) of each specific cell type. This technique will be vital in gaining information upon cellular mechanics when using next-level, multi-cellular *in vitro* strategies.

## Introduction

Understanding the cell type specific response has been shown to be vital in determining the pathology and cellular signalling associated with the onset and progression of disease states, as well as investigating a cells’ role in the development, response and differentiation of tissues *in vivo* ^1^. Single cell and monoculture *in vitro* approaches have previously been shown to be beneficial towards understanding such aspects, particularly within human immunology^2^. Nonetheless, in light of the recent memorandum dictating the replacement of *in vivo* research strategies^3^, significant progress has recently been made in the development of next-level, advanced *in vitro* methodologies^4^, with a particular focus towards multi-cellular systems that combine important cell types responsible for organ-specific homeostasis^5^. Examples of these include models relevant to important regions of the lung^6^, liver^7^, brain^8^ and gut^9^ of the human body. Recently, these co-culture models have been shown as advantageous beyond simple monocultures, both in their ability to realistically elucidate biochemical and biomolecular effects^10, 11^ and, importantly, mimic the cellular interplay that occurs *in vivo* ^12^.

Thus earmarked as valid reduction, as well as potential replacement test systems^13^, intensive efforts are currently directed towards correlating the response metrics observed within such *in vitro* models to that noted using *in vivo* testing strategies^14^. Based on their ability to evolve and imitate the cellular interplay observed *in vivo* ^15^, a major asset for co-culture *in vitro* systems is the possibility to assess and quantify the specific cell type response without need of fixation protocols or specific dissection of the convoluted environment that is human tissue. Such information would further be pivotal in elucidating specific cell type biochemical and biomolecular mechanisms up- or down-regulated following exposure to xenobiotics^16^, as well as allow for clarity in the determination of the specific targeting of (cancer) cells for (new) therapeutic-based approaches^17^.

Yet, to date, progress towards the identification and subsequent quantification of the specific cell type response within multi-cellular *in vitro* models has not been achieved. Thus, here, we report a simple yet effective, reproducible and non-laborious technique based upon multi-colour flow cytometry that can be used to identify the specific cell response, such as oxidative stress, from an established, well studied multi-cellular system consisting of a confluent and tight human lung epithelial tissue layer, as well as two important immune cells (*i.e.* human blood monocyte derived macrophages and dendritic cells)^6^.

## Results

**Figure 1:**
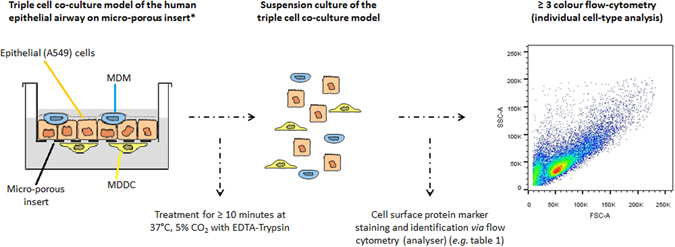
Schematic drawing representing the herein presented methodology. Briefly, an established triple cell co-culture model of the human lung epithelial tissue barrier (consisting of an epithelial cell layer complimented with human blood monocyte derived macrophages and dendritic cells on the apical and basolaterial sides respectively), cultured on a micro-porous membrane insert is detached to form a cell suspension using a reproducible method based upon a short Trypsin-EDTA treatment. After successful detachment, the multi-cell suspension can then be analysed *via* multi-colour flow cytometry (FACS) to gain a perspective upon the status of each specific cell type of the co-culture system.

As illustrated in Fig. 1, specific manipulation of the 3D triple cell co-culture system grown on a micro-porous membrane insert using a short treatment (≤10 minutes) of Trypsin-EDTA enabled the formation of a cell suspension of epithelial cells (*i.e.* adenocarcinoma epithelial type II A549 cell-line) with human blood monocyte derived macrophages (MDM) and dendritic cells (MDDC). Subsequent to obtaining this cell suspension, it is considered that any biochemical or microscopic technique can then be performed in order to gain further, valuable insights in the cellular interplay and effects that occur within such advanced *in vitro* systems following exposure to any form of xenobiotic.

**Figure 2:**
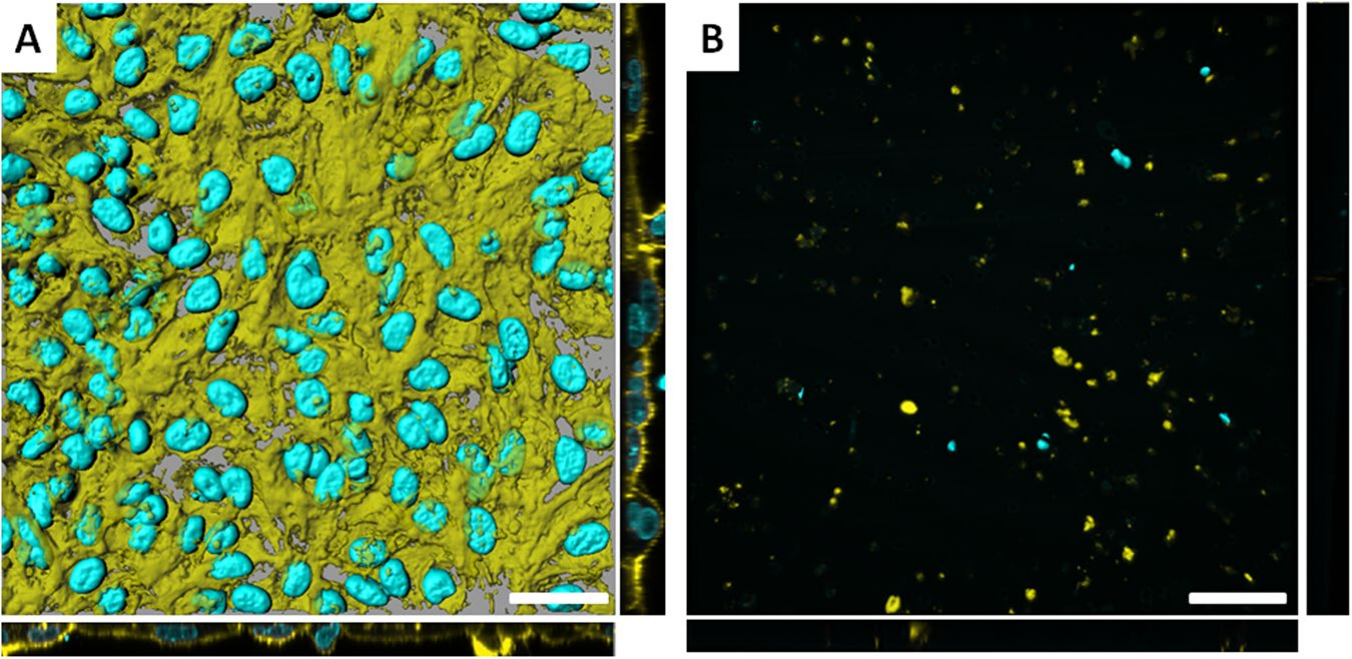
Surface rendered confocal laser scanning microscopy (LSM) images of (**A**) the triple cell co-culture model cultured on a micro-porous membrane insert, and (**B**) a micro-porous membrane insert that originally had the co-culture system cultured upon it and subsequently having been treated with Trypsin-EDTA for ≥10 minutes at 37 °C, 5% CO_2_. Image (**B**) indicates that the Trypsin-EDTA was successful in detaching the majority of the co-culture from the micro-porous membrane insert in order to form a multi-cell suspension. Image A shows the morphology of the co-culture system when grown under normal culture conditions. Both images show staining for the F-actin cytoskeleton (Phalloidin-Rhodamine) and nuclear (DAPI) regions respectively. Scale bars represent 30 µm. In both (**A** and **B**), images show the XY plane in a 3D rendered format (as computed using the software IMARIS®, Switzerland), as well as planar views of XZ and YZ shown in 2D.

Initially, to prove that the method was able to completely detach cells from the micro-﻿porous membrane without causing cell death, within individual wells, the co-culture was either exposed to Trypsin-EDTA or not. Samples were subsequently fixed, and underwent immunofluorescent staining to identify both the cellular F-Actin cytoskeleton and nucleus. As shown in Fig. 2, following the Trypsin-EDTA treatment all cells were completely removed from the micro-porous membrane insert, compared to the co-culture not treated with Trypsin-EDTA. These qualitative, morphological images show that the treatment method was highly effective in detaching the triple cell co-culture model from the micro-porous membrane insert, allowing a cell suspension to be successfully obtained. The images further highlight that the multi-cellular system showed a well-defined monolayer of viable cells, as routinely shown with this co-culture model (*e.g.* Blank *et al.* ^12^) prior to the Trypsin-EDTA treatment.

**Figure 3:**
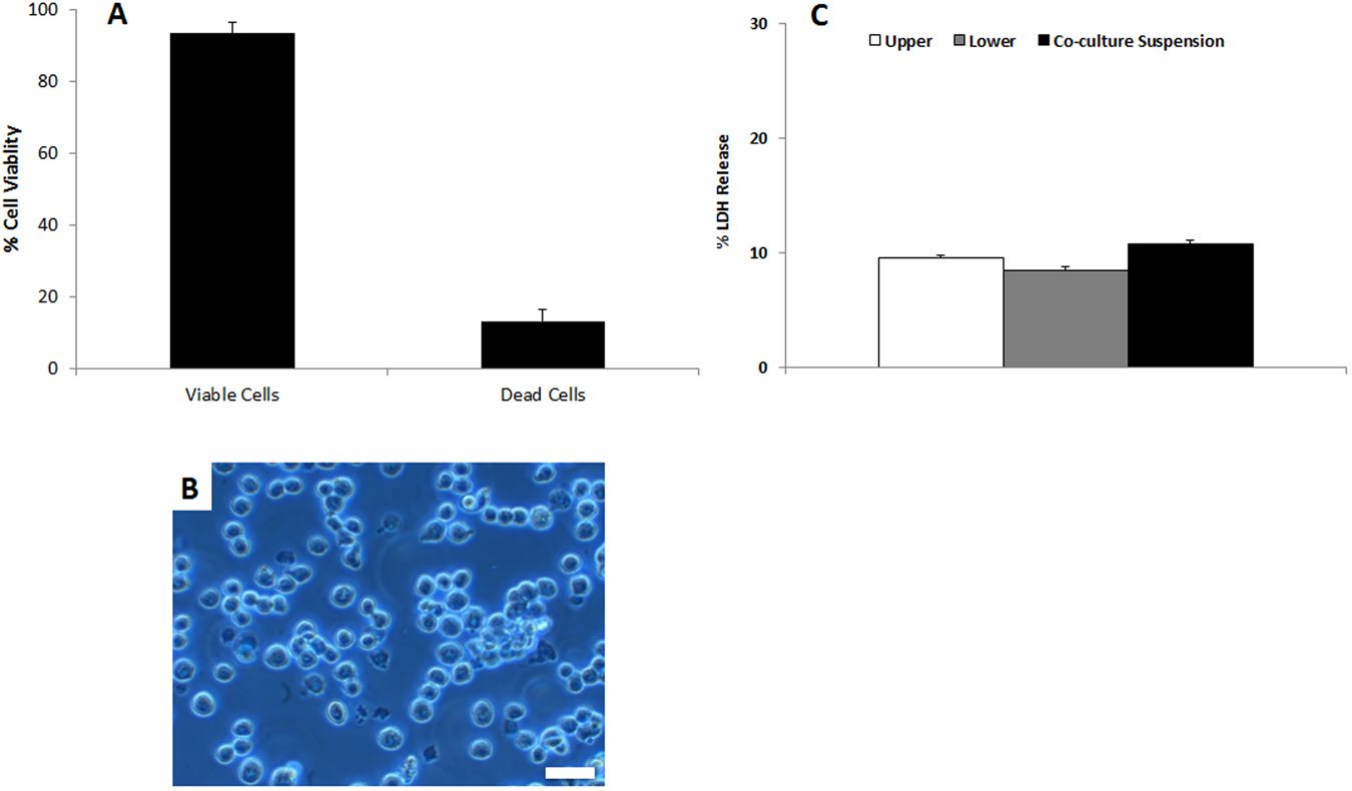
Viability based analytics of the multi-cell suspension. (**A**) Shows the percentage viability of the multi-cell suspension following the Trypsin-EDTA treatment as determined by the Trypan blue assay. (**B**) Shows a representative, 2D light microscopy image of the cell suspension. (**C**) Shows the percentage lactate dehydrogenase (LDH) release of the co-culture when attached to a membrane insert, as well as when in suspension. Specifically, the cell supernatant of the apical (upper) and basolateral (lower) compartments of the co-culture on a micro-porous insert membrane were analysed separately. These data sets were then compared to the cell supernatant of the multi-cell suspension following light centrifugation. In (**A** and **C**), data is expressed is the mean ± standard error of the mean (SEM) (n = 3). In (**B**), the scale bar represents 20 µm.

Subsequent viability analysis of the cell suspension showed that this culture method elicited limited cytotoxicity upon the cell system. Trypan blue analysis indicated, both quantitatively (Fig. 3A) and qualitatively (Fig. 3B) that there was only a 6–8% loss in total cell viability whilst, investigation of the release of the cytosolic enzyme lactate dehydrogenase (LDH) revealed that no significant cytotoxicity occurred. This was achieved by comparing the LDH content of the cell supernatant of the conventionally grown co-culture system on a micro-porous membrane (upper and lower compartment supernatants were analysed as separate entities) against the supernatant of the cell suspension (following centrifugation (0.5 *g* for 5 mins)) (Fig. 3C). Combined, these findings emphasize the feasibility and reproducibility of the method in accomplishing a viable cell suspension of an *in vitro* multi-cellular model.

To further assess the suitability of this method to determine the cell specific response, it was necessary to identify each specific cell type with relevant and appropriate cell surface protein markers. To achieve this, a systematic analysis via single- and multi-colour flow cytometry analysis (FACS) was undertaken.

As a first step, the specific surface markers to label each cell type were optimized using monocultures in regards to antibody concentration and incubation time (SI Table 1). Antibody markers CD11a and CD14 for MDM, Pan-Cytokeratin and E-Cadherin (CD324) for A549 cells as well as CD1c for MDDC were identified by a specific gating strategy that considered the fluorescent signature, size and granularity of the cell population (SI Fig. 1a). From this approach, as determined *via* one-colour flow cytometry, it was observed that all cell surface protein markers elicited a >80% expression frequency (SI Fig. 2A and B) when compared to the unstained control. Interestingly however, CD80, used as an additional marker to characterise MDDC, showed limited binding frequency to the surface of these type of immune cells as a monoculture, with only a ~25% positive frequency (SI Fig. 2B) compared to ~89% for the alternative MDDC marker employed (*i.e.* CD1c) (SI Fig. 2A). Nonetheless, compared to the unstained control, the increase in percentage frequency observed for the specific, direct fluorescence of the CD80 antibody can be considered as effective in differentiating the CD80^+^ population from the CD80^−^ population within the cell suspension.

At this point it is important to note that the data presented for the two immune cells (*i.e.* MDM and MDDC) was obtained following the commonly used culturing method of scraping from the cell culture inserts. As described in the technique for the multi-cellular system, Trypsin-EDTA was used to detach the co-culture from the micro-porous membrane inserts. Thus, in order to control that these important immune cells still exhibited the same degree of phenotype following this chemical-based treatment, a control experiment was performed comparing the scrapping method against Trypsin-EDTA culture. As shown in SI Fig. 3, MDM and MDDC that were cultured using the Trypsin-EDTA protocol showed no significant difference, when compared to the conventional scrapping method, in their ability to express the specific cell surface protein markers for MDM (CD14 and CD11a), and MDDC (CD1c and CD80) in both monoculture and co-culture formats (SI Fig. 3A (monoculture format) and SI Fig. 3B (co-culture format)). It is important to note at this point that for the co-culture suspension samples, due to the specific scatter plot obtained for the co-culture suspension, a further gating strategy adapted from the one previously used for each specific monoculture, and to consider each cell type within the co-culture system was implemented (SI Fig. 1b). Thus, overall, it can be summarized from these findings that Trypsin-EDTA has no impact upon the phenotype of the immune cells used in this study.

**Figure 4:**
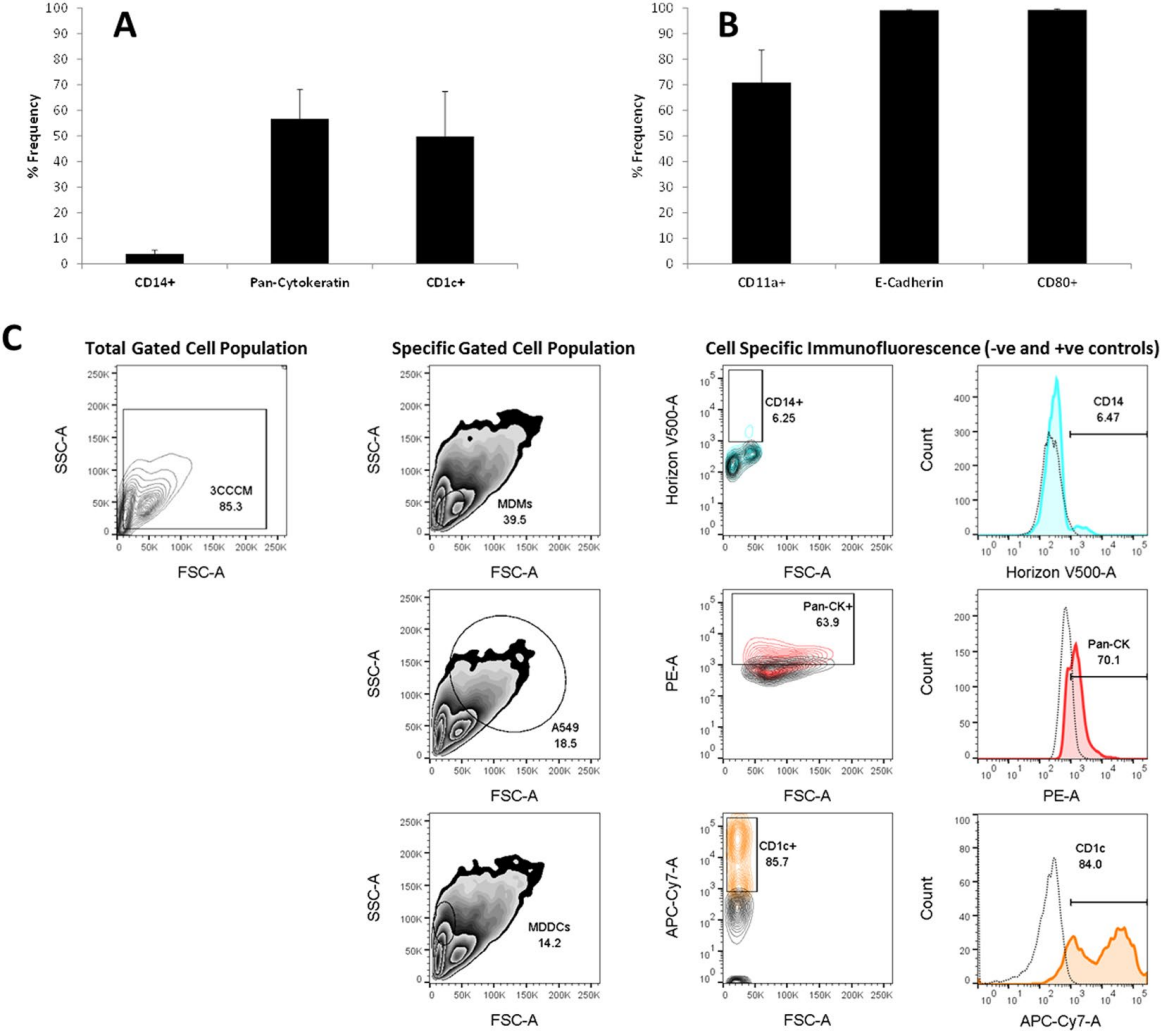
Percentage frequency (%Frequency) of specific surface marker expression upon human blood monocyte derived macrophages (MDM) and dendritic cells (MDDC), as well as A549 epithelial cells when present in the multi-cell model suspension culture. In (**A**) CD14, Pan-Cytokeratin (A549 epithelial cells) and CD1c and (**B**) CD11a (MDM), E-Cadherin (A549 epithelial cells), CD80 (MDDC) show the average %Frequency for the antibody master-mix used (please refer to SI Table 1 for all antibody information). Data was obtained after implementing the specific gating strategy for the co-culture suspension (SI Fig. 1B) (**C**). Samples were analysed *via* three-colour flow cytometry (FACS). All data was analysed using FlowJo (Version 10, TreeStar, USA). In (**A** and **B**) all data expressed is the mean ± standard error of the mean (SEM) of the %Frequency. Experimentation was repeated on three separate occasions in triplicate (n = 3).

After confirming the effectiveness of the specific surface antibodies used in each monoculture, they were assessed, as an antibody master-mix, for their ability to identify each cell of the triple cell co-culture model in the acquired cell suspension *via* three-colour FACS (Fig. 4). In order to identify the specific cell types *via* their fluorescent signature, a specific FACS-based gating strategy was implemented (as described in SI Fig. 1b). Coinciding with their specific fluorescent properties (*i.e.* excitation/emission λ), a master-mix of CD14, Pan-Cytokeratin and CD1c was used to initially define each cell type with the multi-cellular cell suspension (Fig. 4A). As the epithelial cells constituted the greatest number of cells in the suspension, it was unsurprising that Pan-Cytokeratin showed a strong expression within the co-culture suspension (~60% expression). However, for both immune cell types, which showed a ~98% (MDM CD14) and ~89% (MDDC CD1c) expression frequency in monocultures respectively, a significant loss in expression to 5% (MDM CD14) and 50% (MDDC CD1c) was observed compared to the negatively stained control (*i.e.* co-culture suspension treated with *complete medium* only) (Fig. 4A). Similarly, within another master-mix cocktail, the marker for MDM, CD11a, also showed a loss in expression frequency, with only 70% observed in the co-culture suspension compared to ~98% in MDM monocultures (Fig. 4B). CD80 (MDDC) further exhibited only a *ca.* 90% expression within the co-culture suspension. Although significant, this is only attributable to a loss of *ca*. 8% from the MDDC monoculture scenario. Again, contrasting with the immune cell types, the epithelial marker, E-cadherin, as seen with Pan-Cytokeratin, showed a higher expression compared to the immune cells, with a %Frequency of 92–98% (Fig. 4B). Interestingly, E-cadherin showed a 90% Frequency expression within the co-culture compared to the Pan-Cytokeratin marker, highlighting both as suitable in identifying the epithelial cell cache.

**Figure 5:**
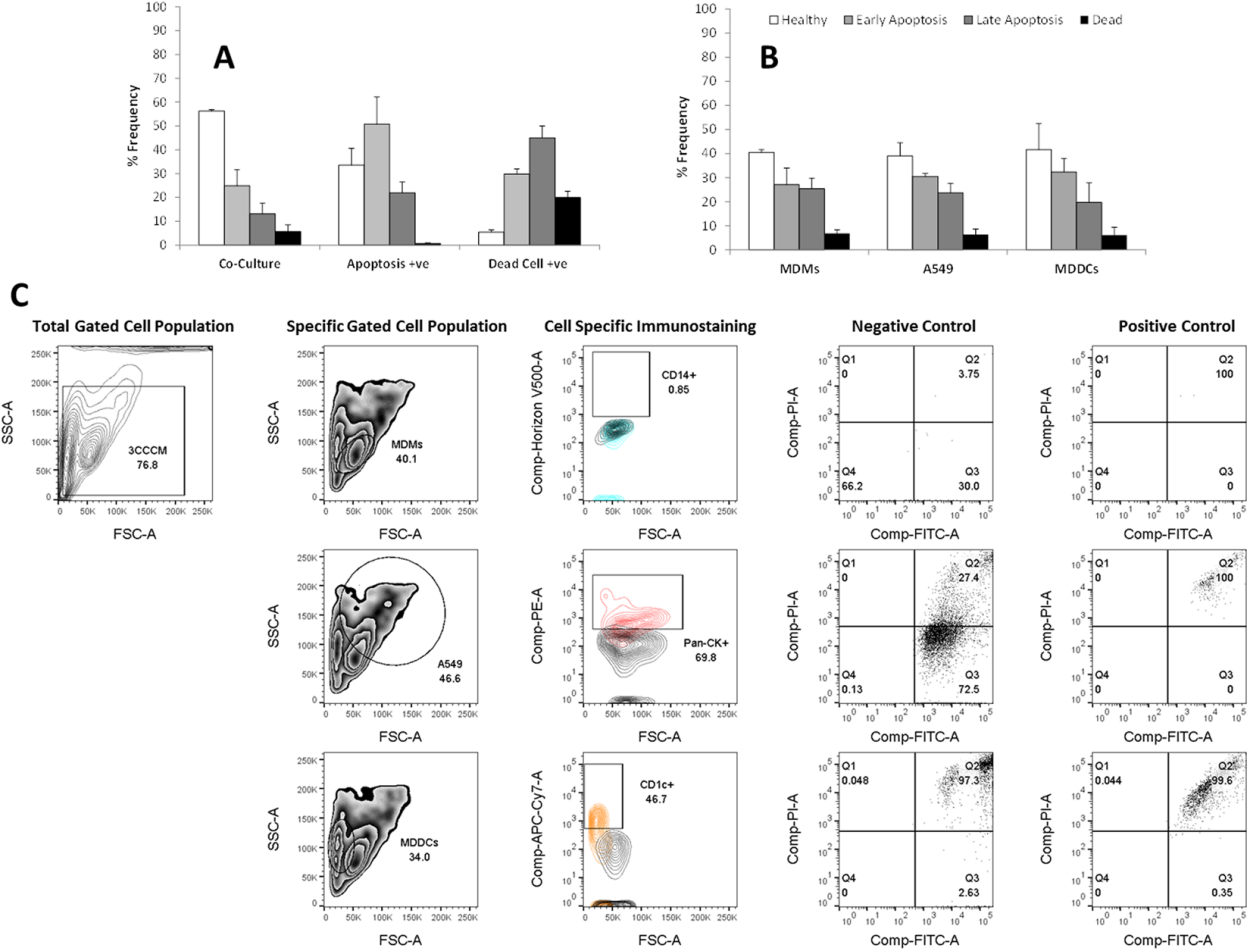
Investigation of the ability for the herein presented method to obtain a cell suspension of the 3D *in vitro* triple cell co-culture model to incite cell death, as determined with the Annexin V assay. Following detachment from a micro-porous membrane insert via EDTA-Trypsin treatment the level of cell death (*i.e.* either apoptosis or cell death (*e.g.* necrosis)) was determined for (**A**) the total cell suspension *via* two-colour flow cytometry (FACS), or (**B**) for each specific cell type of the co-culture system *via* five-colour FACS. In both (**A** and **B**), data shows the viability of the co-culture following 24 hours exposure at 37 °C, 5% CO_2_ to either *complete medium* (negative control), Camptothecin at [0.002 mg/mL] (positive apoptosis control) or extreme freezing (−80 °C for 30 minutes) (positive dead cell (*i.e.*necrosis) control). After implementing the specific gating strategy for the co-culture suspension (SI Fig. 1B) (**C**) indicates the gating strategy used to deduce the viability status of the cell cultures was defined as healthy, early apoptotic, late apoptotic or dead. All data was analysed using FlowJo (Version 10, TreeStar, USA). In (**A** and **B**) all data expressed is the mean ± standard error of the mean (SEM) of the %Frequency. Experimentation was repeated on three separate occasions in triplicate (n = 3).

After confirming the ability to easily identify each specific cell type within the multi-cell suspension, additional viability analysis was performed in the manner of the Annexin V assay^18^, which determined the specific cell death status of each cell type of the co-culture *via* FACS (Fig. 5A–C). By initially incorporating two-colour FACS analysis, it was possible to differentiate between total number of cells undergoing apoptosis, and other forms of cell death (*e.g.* necrosis) within the cell suspension. When exposed to *complete medium* only over a 24 hour period, two-colour flow cytometry of the entire co-culture showed a frequency of 55% as the healthy cell population compared to both 22% and 14% early and late apoptotic cell populations respectively (Fig. 5A). It was also observed that 9% of the cell population was considered dead cells (*i.e.* not apoptotic, but suggestive of necrotic cell death) (Fig. 5A). Compared to the positive control for apoptosis (*i.e.* Camptothecin), of the gated population, it was observed that the frequency of healthy cells reduced to 33%, whilst the early apoptotic cell population increased from 22% to a 51% expression frequency (Fig. 5A). The late apoptotic cell population increased to 24% from 14% (negative control) (Fig. 5A). Reasoning for the significant differences between the early and late apoptotic cell populations can be attributed to the time-point studied (*i.e.* 24 hours). Furthermore, to prove that the method described herein did not cause only apoptosis, but also any other major cell death pathway (*i.e.* necrosis) the positive control for dead cells showed a 25% population for dead cells compared to 3% for live cells (Fig. 5A). It is also interesting to note that whilst this data set indicates that the positive control for dead cells worked adequately, the percentage frequency of both early (30%) and late apoptosis (50%) suggests this methodology is not completely specific for non-apoptotic cell death. This data therefore supports the previous total cell analysis (Fig. 3) that showed the here presented culture method incites limited cytotoxicity and little adverse impact upon cellular viability.

In order to support the hypothesis that this culture method allows for the specific determination of cell type effects within a complex, multi-cell suspension, further investigation of the viability of the co-culture model was analysed on the single cell type level *via* five-colour FACS following exposure to *complete media* only. Due to the properties of the Annexin V fluorophores, CD14, Pan-Cytokeratin and CD1c were used to specifically identify the MDM, epithelial cell (A549) and MDDC populations. Analysis across the different cell types indicated that this novel technique implemented no deleterious impact upon any one specific cell-type (Fig. 5B). The findings show that there are no significant differences between the viability of the different cell types within the suspension of the co-culture itself. In fact, for MDM and MDDC, as well as A549 cells the healthy cell population was observed to be between 40–45%, with a 25–35% early and late apoptosis respectively as well as <10% dead cells (Fig. 5B). It is also important to note that this data supports that previously shown (Fig. 3). Thus, from the data presented, it is possible to state that herein presented culturing technique to obtain a multi-cell suspension from an *in vitro* multi-cellular model incites limited cytotoxicity/impact upon cellular viability, as well as, that it is possible, for the first time, to efficiently and effectively identify each specific cell type within the model, and the status of each specific cell type within it.

**Figure 6:**
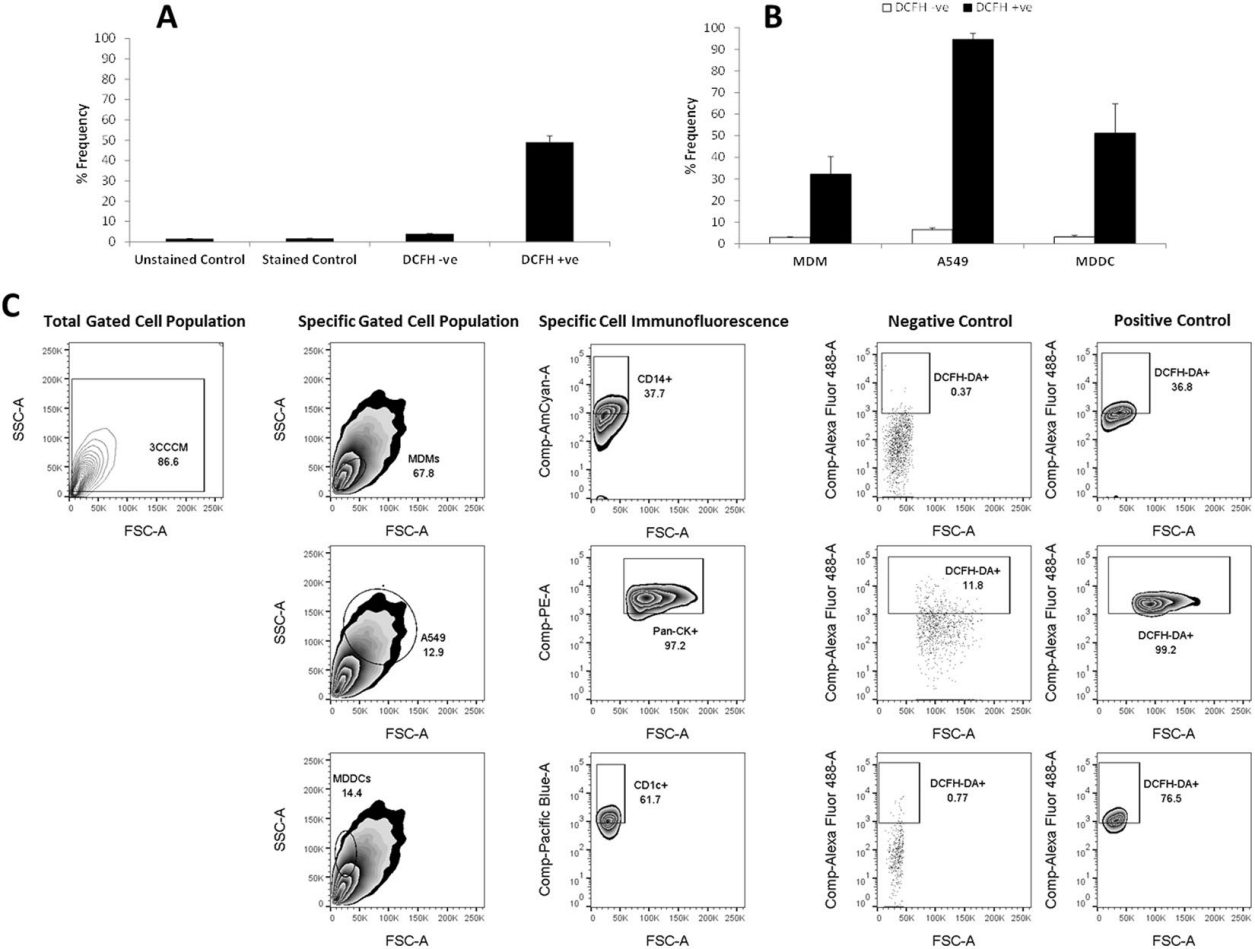
Investigation of the ability to detect the presence of reactive oxygen species (ROS) using the fluorescent probe 2′,7′-dichlorfluorecein-diacetate (DCFH_2_-DA) (*i.e.* the DCFH-DA assay) in the 3D *in vitro* triple cell co-culture of the human lung epithelial tissue barrier following detachment from micro-porous membrane insert *via* EDTA-Trypsin treatment. The ROS response was measured for (**A**) the total cell suspension *via* one-colour flow cytometry (FACS), or (**B**) for each specific cell type of the co-culture system *via* four-colour FACS. In both (**A** and **B**), data from one- and four-colour FACS shows the overall ROS response in the co-culture suspension following 4 hours exposure at 37 °C, 5% CO_2_ to either *complete medium* (negative control) or *tert*-Butyl Hydrogen Peroxide (*t*BHP) at [0.04 mg/mL]. Following the exposure period, the co-culture cell suspension was treated with 1:10 dilution of 1 mM DCFH-DA (488 nm). A DCFH-DA treated control (DCFH-ve) was also performed to denote any non-specific fluorescence within the sample. After implementing the specific gating strategy for the co-culture suspension (SI Fig. 1b) (**C**) indicates the gating strategy used to understand a negative and positive response from the multi-cell suspension using the DCFH-DA assay. All data was analysed using FlowJo (Version 10, TreeStar, USA). Data expressed is the mean ± standard error of the mean (SEM) of the %Frequency. In (**A** and **B**) all data expressed is the mean ± standard error of the mean (SEM). Experimentation was repeated on three separate occasions in triplicate (n = 3).

Although it was possible to determine the specific viability status of each cell type of the co-culture system, it was important to further note if the method can allow for the assessment of sub-lethal biochemical and biomolecular effects, as this will allow for important investigation of mechanistic-toxicology effects at an *in vitro* level. In order to deduce this investigation of the production of reactive oxygen species (ROS) was performed. ROS analysis was chosen since this endpoint is an essential and important endpoint within all toxicological-based research, as well as is a major biochemical driver regarding the onset of any adverse biological response, such as genotoxicity^19^. ROS response was measured using the commonly used, semi-quantitative technique that incorporates the fluorescent probe 2′,7′-dichlorfluorecein-diacetate (DCFH_2_-DA) (*i.e.* the DCFH-DA assay)^20^. In principle, the method is based upon the hydrophobic molecule DCFH_2_-DA, which oxidises when in the presence of esterases to form the highly fluorescent dichlorofuorescin (DCF)^21^. To study this, the co-culture was either treated with *complete medium* or a known oxidant^20^, *tert*-Butyl Hydrogen Peroxide (*t*BHP) at [0.04 mg/mL] for 4 hours at 37 °C, 5% CO_2_. Initially the total cell effect was analysed *via* one-colour FACS (Fig. 6). As shown in Fig. 6 the ROS production in the entire co-culture showed a significant increase in expression frequency following exposure to *t*BHP compared to *complete medium* only over a 24 hour period (DCFH +ve sample showed a > 50% Frequency increase above negative, stained and DCFH −ve samples). Therefore, it was possible to measure a sub-lethal response within the multi-cell suspension dependent upon the specific chemical treatment. Subsequent, cell specific analysis was then performed using an antibody master-mix as previously described with the Annexin V assay, albeit with only four-colour FACS. Investigation showed that both immune cell types elicited a significant decrease in ROS production compared to the A549 cells (~95% compared to ~55% (MDDC) and ~35% (MDM) (Fig. 6). This effect is surprising due to the immune cell function during oxidative stress^19^. However, interestingly the MDDC showed a heightened ROS production compared to the MDM, thus suggestive that the intricate cell-to-cell interplay is possibly evident, even within the multi-cell suspension and not only when constructed on the micro-porous membrane insert (Fig. 6).

**Figure 7:**
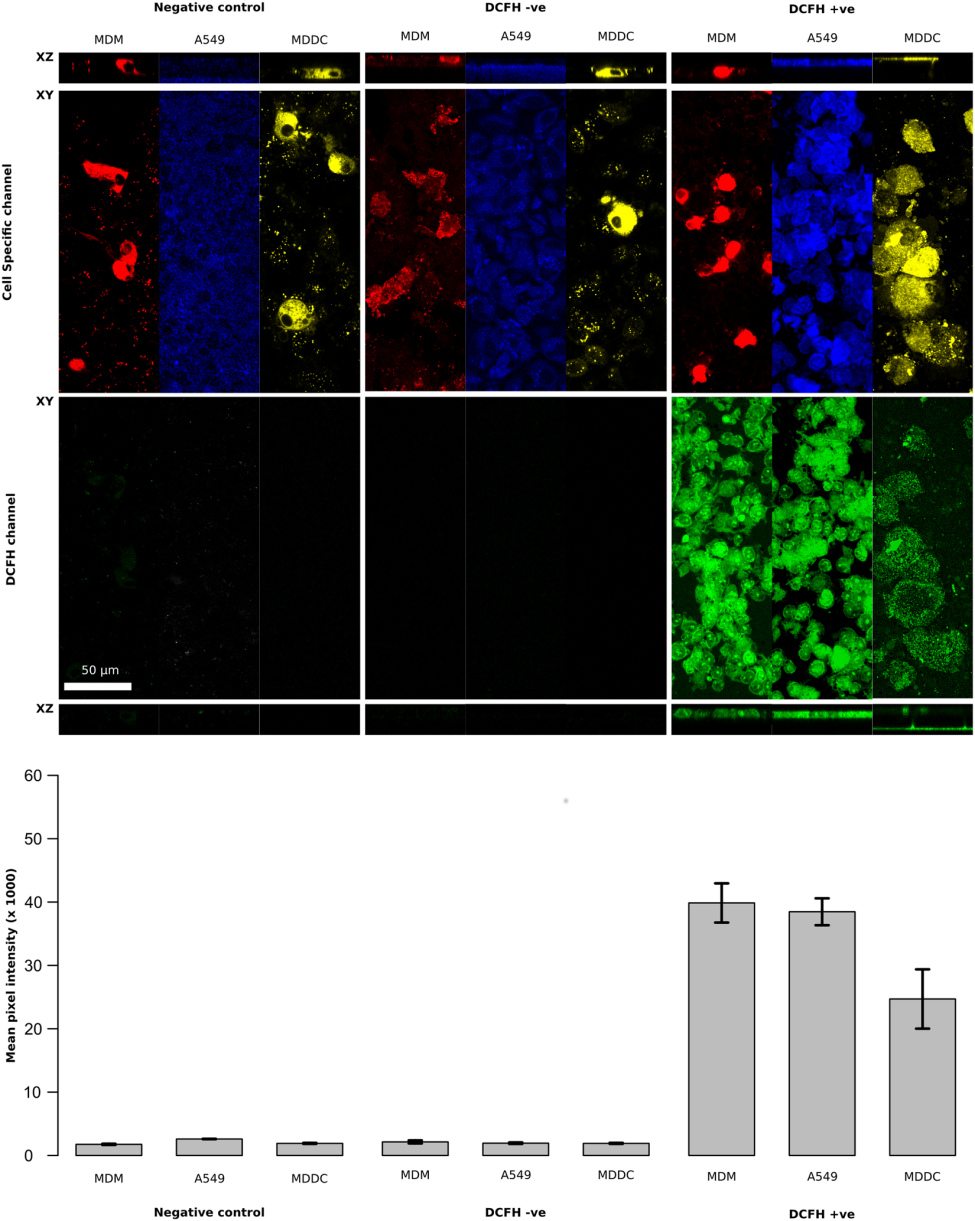
Ability to detect the production of reactive oxygen species (ROS) using the fluorescent probe 2′,7′-dichlorfluorecein-diacetate (DCFH_2_-DA) (*i.e.* the DCFH-DA assay) in the 3D *in vitro* triple cell co-culture of the human lung epithelial tissue barrier *via* live confocal laser scanning microscopy (LSM). To denote each specific cell type, live cell dyes (Vybrant Multicolor, Cell-Labeling Kit (Molecular Probes, Switzerland) were used to identify both the human blood monocyte derived macrophages (MDM; DiL (549/565 nm)) and dendritic cells (MDDC; DiD (644/665 nm)). A Cell Tracker Violet live dye (Molecular Probes, Switzerland) was then used to define the A549 epithelial cell layer apart from the specifically stained immune cell caches. ROS production was subsequently determined in the co-culture system following 4 hours exposure to either *complete medium* or *tert*-Butyl hydrogen peroxide (*t*BHP) at [0.04 mg/mL]. Following the exposure period, the co-culture was stained with 1:10 dilution of 1 mM DCFH-DA (488 nm). A DCFH-DA treated control (DCFH-ve) was also performed to denote any non-specific fluorescence within the sample. Representative images of each specific cell type under the same exposure conditions are shown the XY and XZ planar views in 2D. Semi-quantitative analysis shown in the lower bar-charts was determined using the cell specific marker and calculated on a single cell level.

In order to confirm the results from the FACS analysis, laser scanning confocal microscopy (LSM) was utilized using the co-culture model when attached to the micro-porous insert membrane. Specifically, this was chosen as a supportive method as it is possible to identify the single cell effect under microscopic analyses, due to the ability to label each cell type with a specific fluorescent marker, just as with FACS analysis. However, it is important to point out that *via* this technique it is not possible to get an adequate (semi-)quantifiable analysis, such as with FACS or other methods (*e.g.* polymer-chain reaction (PCR)) which the herein culture technique has been shown to allow for (as reported above in Fig. 6). Thus, a correlative analysis was undertaken between the ROS analysis performed with four-colour FACS compared to live LSM. As shown in Fig. 7, all cell types showed a distinct level of ROS production when exposed to *t*BHP at [0.04 mg/mL] for 4 hours at 37 °C, 5% CO_2_ in the presence of the DCFH-DA (*i.e.* DCFH +ve). Importantly, both the negative control (*complete medium* only) and the DCFH −ve samples showed no ROS response in these scenarios, thus supporting the previous four-colour FACS analysis. Nonetheless, the semi-quantitative analysis performed upon the live LSM data *via* the mathematical approach described in the online methods section and shown in the form of bar-charts in Fig. 7 (which used single cell analysis of the fluorescent properties on the DCFH +ve channel (FITC (488 nm)), showed the following hierarchical ROS effect response; MDM > A549 > MDDC. This finding is in contrast to the four-colour FACS analysis that showed a response effect of A549 > MDDC > MDM in terms of their ROS production. Thus, in order to comprehend and compare adequately across methods, the ROS response measured *via* FACS was also determined on a single cell level. Through dividing the %Frequency response with the number of gated events, it was possible to deduce the average, single cell ROS response *via* FACS. As shown in Fig. 6D, it was observed that at the single cell level within the specific cell type analyses that the ROS response was similar, albeit not identical, in terms of the ROS production trend across the cell types.

## Discussion

Determining the specific cell type response can provide essential information towards the onset and progression of disease states, as well as comprehend the efficiency and effectiveness of therapeutic care (*i.e.* drug targeting to specific cell types). With the need for alternative *in vitro* systems on the constant increase, the use of advanced *in vitro* multicellular systems as advantageous, model systems has been heightened in recent years. Thus, to provide a further dynamic dimension towards such *in vitro* co-culture systems, that have been shown to supersede monocultures when analysing beyond a simple live/dead investigation^11^, the objective of this study was to establish and present a reliable, effective and straight-forward method that could be implemented to study, and importantly quantify, the specific cell type response as a progression beyond current practices (*i.e.* microscopy based methods) that can only qualify such specific cell type effects.

In the present study, the data presented highlights that the simple approach of detaching an established multi-cellular system of the human lung epithelial tissue barrier from a micro-porous insert membrane was reproducible, and did not induce any form of significant cell death to the total cell culture, or, after successful identification of each cell type that constitutes the co-culture model *via* multi-colour FACS, any adverse effects to the viability of each specific cell type. Further to this, the method was proven to be able to detect, and (semi-)quantify the sub-lethal, biochemical effects (*i.e.* ROS production) at a single cell type level within the multi-cellular model.

Although it was possible to identify each specific cell type of the multi-cellular model under suspension conditions *via* multi-colour FACS, it is important to highlight that a significant loss in %Frequency was observed with both immune cell types (MDM and MDDC) in the co-culture suspension compared to the monoculture format. This noticeable effect could be attributed to the fact that the immune cells within the co-culture suspension undergo treatment with EDTA-Trypsin, which is not the normal cell detaching technique for primary isolated immune cells from human blood (*i.e.* the scrapping method is most commonly used). However, as shown in SI Fig. 3, there was no effect upon the expression of the surface markers used to identify the immune cells across the mono- or co-culture formats, or the different culturing techniques. Therefore, the findings could be suggestive of (i) a lack of binding efficiency of the surface markers chosen to identify the immune cells of the 3D co-culture model when in suspension (*i.e.* there was a lack of available receptors for each surface protein marker on the immune cells in the co-culture suspension), (ii) the lack of any immune cells present, (iii) a loss in cell in cell viability (*i.e.* cell death) or (iv) a differentiation of the cells in the co-culture environment resulting in down-regulation of the surface markers. Yet it is also apt to consider that none of the above hypotheses are actually correct in terms of the observations presented. Instead, it is important to note the relative cell number identified and analysed. Firstly, the significant differences in the expression frequencies of the two immune cell types between monocultures and co-cultures can easily be attributed to the difference in initial seeding density or possible cell-cell interactions which can lead to an altered differentiation. For both MDM and MDDC monocultures they were originally seeded at 1 × 10^6^ cells/mL, whereas in the co-culture, upon micro-porous inserts, MDM were seeded at 0.5 × 10^5^ cells/mL and MDDC at 2.5 × 10^5^ cells/mL. Notably, for the epithelial cells, this issue was not observed since both mono- and co-culture scenarios cells were cultured at 0.5 × 10^6^ cells/mL, although interestingly the marker E-cadherin showed a higher expression frequency in the co-culture suspension than with the A549 monoculture suspension (as well as beyond that seen for Pan-Cytokeratin in either cell culture format). E-cadherin is a calcium-dependent cell adhesion molecule that is important in the integrity of epithelial cell adherent junctions^22^, as well as the suppression of tumour formation^23^. This finding could therefore be attributed to the fact that when combined with immune cells the A549 cells show a greater cell-cell adhesion than in monocultures following seven days culture.

Secondly, the technique herein presented obtains a cell suspension of the co-culture from the model cultured on a micro-porous membrane. Previously, Blank *et al.* ^12^ reported the number of cells within the co-culture (~50’000 MDM: ~500’000 epithelial cells; 250’000 MDDC), reporting a 1:10:5 dilution of MDM:A549 cells:MDDC (based on MDM number). This ratio however is relative to the seeded density and does not consider the cell number upon direct experimentation with the co-culture system. It was further presented by Brandenberger *et al.* ^24^ therefore, that the cell:cell ratio was 1:40:0.7 (based again on MDM number) when counted during experimental conditions. This finding particularly highlights that due to the use of primary immune cell cultures, the accepted ‘gold-standard’ within *in vitro* based research (*i.e.* for drug design and delivery)^25^, there is a constant variability and quality of the cells used that has to be significantly considered throughout all experimentation. In the present study however, following the procedure to obtain a cell suspension it was observed that from the specific gated population of the co-culture 25% were MDM, 46% A549 cells and 29% MDDC, indicating a ratio of 1:1.8:1.1 (based on repeated measurements contusive with the analysis presented in Fig. 4). Therefore, attention must be directed towards the experimental conditions. In this regard, it should be considered that for analysis only a density of 1 × 10^6^ cells/mL were analysed and not the entire yield of the suspended co-culture itself. From the co-culture analysis of the Annexin V assay results, it can be seen that viable cells were only seen at 55% Frequency, and that cell death, through whichever pathway (*e.g.* apoptosis or necrosis), was relative for the remainder of the gated cell population. Thus, these aspects could be considered as significantly accountable for the differences in the %Frequency observed for the immune cell types in the co-culture suspension compared to their monoculture format.

On another note, it is prudent to point out the differences between the FACS and LSM analyses regarding the measured ROS response. Although not completely identical, at the single cell type level it was shown that the methods and culture conditions complemented one another well (*i.e.* showed the same trend of effect across the specific cell types), indicating that the cellular effect of the co-culture is not impeded by the ability to form it from a static, adherent culture to a cell suspension. It is also important to note that detection of fluorescent signals with LSM can cause bleaching, since the cells are not unfixed but observed under live conditions and also (possibly) inform a 3D perspective, implicating a longer laser time. Bleaching effects are, however not associated with FACS. Nonetheless, it should be highlighted that there is a high level of variation across the cell types, indicating that although it is possible to show that there is a potential driving effect by one specific cell type, cell heterogeneity, which has recently been shown to have a significant impact upon measured cellular response^26^ within the cultures is vast. This, as highlighted before, is due primarily to the primary isolated immune cell cultures which exhibit high variability due to donor variation.

In perspective of the currently presented methodology, as an outlook towards its use within different *in vitro* based disciplines, it must be emphasized that multi-colour FACS is merely one avenue of quantification-based analysis that can be performed. Additional biochemical and microscopy based protocols (*i.e.* high content screening microscopy) could be undertaken in order to decipher and elucidate further the specific cell type effect across a number of different toxicological-based endpoints (*e.g.* gene-expression *via* PCR and DNA damage *via* COMET assay). This is important to note, since despite FACS analysis being needed to confirm the applicability and effectiveness of the presented method, as well as FACS-based cell sorting required to obtain each cell type in order to undertake such analyses as *e.g.* PCR, this (semi-)quantitative technique is only suitable for specific endpoints (commonly based upon fluorescence) which does not all for the complete deduction of the specific cell type effect due to a variety of background factors (*e.g.* number of cells analysed, specificity of cell identification).

In conclusion therefore, the herein described methodology is an effective approach towards determining the cell specific response to any xenobiotic when using multi-cellular models *via* multi-colour FACS analysis. This method will offer new opportunities in the field of toxicology research as well as drug design and delivery. It is proposed that this technique will allow for an important analytical understanding to be gained of cell type specific behaviour within advanced *in vitro* cell systems following exposure to drugs or any form of foreign object(s).

## Online Methods

### Chemicals and Reagents

All chemicals and regents were purchased from Sigma-Aldrich (Switzerland), unless otherwise stated.

### Cell Culture

#### Monocultures

Monocultures of human blood monocyte derived macrophage (MDM) and dendritic (MDDC) cells were cultured as previously described by Rothen-Rutishauser *et al*.^6^ and Lehmann *et al*.^27^, with an additional CD14^+^ magnetic bead separation (CD14^+^ isolation beads, MACS Miltenyi Biotech, Bergish Gladbach, Germany; Cat. No.: 130–050–201), as reported by Steiner *et al*.^28^. Both immune cell types were seeded at a density of 1 × 10^6^ cells/mL in 6-well plates (3 mL/well) for a period of 7 days at 37 °C, 5% CO_2_. To promote specific maturation, MDM were cultured with macrophage colony stimulating factor (M-CSF) ([10 ng/mL]), whilst MDDC were culture with interleukin (IL)-4 and granulocyte macrophage colony-stimulating factor (GM-CSF) (both at [10 ng/mL]). In addition, A549 adenocarcinoma type II-like epithelial cells (ATCC, USA) were cultured in 75 cm^2^ cell culture flasks as previously described^6, 27^. The A549 cells were seeded at 0.5 × 10^6^ cells/mL upon BD Falcon^TM^ micro-porous inserts (high pore density PET membranes with a growth area of 4.2 cm^2^ and 3.0 μm pore size (Cat #: 353091); Becton Dickinson AG, Allschwil, Switzerland) (2 mL of cell suspension added to the upper chamber; 3 mL supplemented cell culture medium added to the lower chamber) in 6-well plates for 7 days to enable a tight-epithelial layer to form. For all experimentation passage #’s 5–25 of the A549 cell cultures were used. All cell types were cultured using Roswell Park Memorial Institute (RPMI) 1640 cell culture medium supplemented with 10% fetal calf serum (FCS), 1% penicillin/streptomycin (P/S) and 1% L-glutamine (L-G) (both at [10’000 µg/mL]) (Gibco, Switzerland) (*hereby referred to as* ‘*complete medium*’).

#### 3D Triple Cell Co-culture Model of Human Lung Epithelial Tissue Barrier

The 3D *in vitro* model of the human lung epithelial tissue barrier previously described^6, 15^ was used. Briefly, this model consisted of a layer of A549 epithelial cells cultured upon the same micro-porous membrane insert in 6-well plates as described above. MDM were then cultured on the upper chamber of the insert (*i.e.* apical side of the co-culture model) and MDDC on the lower side (*i.e.* basolaterial side). A total of 7 days prior to the construction of the co-culture model, the A549 cells were cultured at a density of 0.5 × 10^6^ cells/mL at 37 °C, 5% CO_2_ precisely as described above. At the same time, MDM and MDDC were isolated from human buffy coat obtained from the Swiss National Blood Donation Centre (Bern), as described above. After the culture period, MDM were seeded at a density of 50 × 10^3^ cells/insert on the apical side and MDDCs at a density of 25 × 10^4^ cells/insert on the basolaterial side of the insert. The triple cell co-culture was subsequently incubated for 24 hours at 37 °C, 5% CO_2_ for cell-cell habituation with 2 mL and 3 mL cell culture media in the upper and lower chambers respectively. Following this culturing period, the 3D co-culture model was then available for experimentation over a period of 72 hours. All cell culture was performed using *complete medium*.

### Cell Suspensions

#### Monocultures

To obtain a cell suspension of both MDM and MDDC, immune cells were gently scraped from the 6-well plates and then collected. For the A549 epithelial cell-line, cells were treated with 0.5 mL EDTA-Trypsin for 5 minutes at 37 °C, 5% CO_2_. A total of 0.5 mL of *complete medium* was then added following the incubation period to stop the reaction of the EDTA-Trypsin and the cell suspension was then collected following gentle aspiration of the cell cultures. For all cell types, each cell suspension was initially centrifuged at 0.5 g for 5 minutes and the cell pellet then re-suspended in 1 mL *complete medium*. A cell count was then performed using Trypan blue (1:5 dilution) to determine the viable cell fraction. Cells were then suspended within Falcon tubes for flow cytometry (*i.e.* fluorescence-activated cell sorting (FACS)) (Sterile ‘snap cap’ 5 mL tubes (Cat #: 35204), BD Biosciences, Switzerland) at a density of 1 × 10^6^ cells/mL, in preparation for analysis via flow cytometry.

#### 3D Triple Cell Co-Culture of the Lung Epithelial Tissue Barrier

To achieve a cell suspension of the co-culture model, it was initially washed twice with 1 M phosphate buffered saline (PBS) prior to treatment with EDTA-Trypsin (0.5 mL in the upper chamber and 1.5 mL in the lower chamber) for ≤10 minutes at 37 °C, 5% CO_2_. Following the incubation period, equal volume of *complete medium* to EDTA-Trypsin was added to the co-culture in order to negate the effect of the EDTA-Trypsin upon the cell culture. Subsequently, on both the apical and basolateral side of the insert-membrane, cells were gently scraped under aspiration and then collected. Cells were washed once with PBS *via* centrifugation at 0.5 g for 5 minutes and then re-suspended in 1 mL *complete medium*. A cell count was then performed using Trypan blue (1:5 dilution) to determine the viable cell fraction. Cells were then suspended within Falcon tubes for flow cytometry (*i.e.* FACS) (Sterile ‘snap cap’ 5 mL tubes (Cat #: 35204), BD Biosciences, Switzerland) at a density of 1 × 10^6^ cells/mL, in preparation for analysis *via* flow cytometry.

#### Cell Morphology

To assess the ability for the presently described method to detach the co-culture system from the micro-porous membrane insert, confocal laser scanning microscopy was used to confirm the presence of cells on the membrane insert. To achieve this, the triple cell co-culture was cultured on two separate micro-porous membrane inserts. One membrane insert was exposed to the Trypsin-EDTA method as described above, whereas the other was treated with *complete medium* only for the same time and under the same environmental conditions. After the exposure period both membrane inserts were fixed with 3% paraformaldehyde in PBS for 15 minutes at room temperature. After fixation, the samples were washed x3 with PBS and then treated with 0.2% Triton X100 in order to permeablise the cell membrane prior to immunofluorescent staining. Samples were then washed x3 with PBS and stained with Phalloidin-Rhodamine (to identify the F-actin cytoskeleton) using a 1:50 dilution in PBS, as well as 4′,6-diamidino-2-phenylindole (DAPI) (to identify the cell nuclei) at a 1:100 dilution in PBS. Samples were stained for 45 minutes in the dark at room temperature. Following the staining period, samples were washed x3 with PBS and mounted onto glass microscope slides using Glycergel (Dako, USA) and imaged using an inverted LSM 710 Meta (Carl Zeiss, Germany) using a Plan-Apochromat 63x/1.4 lens (NA = 1.3) with 0.3 µm z-stacks to enable the spatial investigation in 3D. Images are shown with the XY plane in a 3D rendered format (as computed using IMARIS®, Switzerland), as well as planar views of XZ and YZ shown in 2D in order to confirm the presence, or absence of cells (*i.e.* cellular layer) attached to the membrane insert.

### Cell Viability

#### Trypan Blue Assay

To further assess the viability of the cell suspension obtained through the here presented method, the suspension was assessed *via* the Trypan blue method. Following the culture method, and using a Trypan blue dilution (1:5), the viable cell fraction was assessed using conventional light microscopy (Motic, AE2000 Inverted Microscope Motic Deutschland GmbH, Wetzlar, Germany) using a 40x magnification. This assay was repeated on three separate occasions (n = 3). On each occasion, 250 cells were counted and the viable cell percentage determined.

#### Lactate Dehydrogenase Release

Cell viability was further determined *via* the level of released lactate dehydrogenase (LDH) from the cell suspension. As a negative control, the LDH release from the triple cell co-culture as cultured upon a membrane insert was also assessed. For each scenario, the cytosolic enzyme LDH was measured in the cell supernatant to obtain an impression as to the permeability/damage of the cellular membrane. In terms of the co-culture on the micro-porous membrane, supernatants were collected from the upper and lower compartments as previously described by Clift *et al.* ^11^. To assess the LDH release in the co-culture suspension, cells were centrifuged at 0.5 g for 5 minutes at 24 °C (*i.e.* room temperature). The supernatant collected from this sample was then analysed for its LDH content. As a positive control, 0.2% Triton X100 was used. LDH levels were determined using a diagnostic kit following the manufacturer’s guidelines (Roche Diagnostics, Switzerland). Data is expressed as the percentage of LDH release compared to the positive control (0.2% Triton X100). This assay was repeated on three separate occasions in triplicate (n = 3).

#### Immunostaining for Specific Cell Surface Protein Markers via FACS

To confirm the ability to resolve each specific cell type with a series of different cell surface protein markers for monoculture and co-culture systems. Before cells were labelled with specific antibodies, cells were incubated with Fc-block receptor (Miltenyi Biotech (Cat #: 130-059901), Switzerland) for 10 minutes in order to inhibit Fc-receptor binding of specific antibodies. The cell suspension was then washed x1 with PBS *via* centrifugation at 0.5 g for 5 minutes at 4 °C. The cell pellet was then re-suspended in 400 µL FACS buffer ((PBS supplemented with 1% bovine serum albumin (BSA) and 0.1% sodium azide (NaN_3_))) and then stained with the specific cell surface protein marker of interest. For all specific protein markers and dilutions used, as well as the fluorescent characteristics of each antibody please refer to SI Table 1. Samples were incubated at 4 °C for 30 minutes. Following the immunostaining period, samples were washed x1 with PBS *via* centrifugation at 0.5 g for 5 minutes at 4 °C. The cell pellet was then re-suspended in 400 µL FACS buffer and analysed by either one- or three-colour FACS. The negative control for all sample analysis was either an unstained cell monoculture (MDM, MDDC or A549 epithelial cells) or co-culture suspension exposed to *complete medium* only. Samples were analysed using two-colour FACS (LSR Fortessa (3 laser, 4-blue-2-red-2-violet (405 nm (violet); 488 nm (blue); 640 nm (red)) BD Biosciences, Basel, Switzerland)). Fluorescent signals were collected in logarithmic mode (4 decade logarithmic amplifier) and cell numbers per channel in linear mode. To identify each cell population, an electronic gate was placed around the side- and forward-scatter modes with 30’000 gated events acquired for each sample. The fluorescent amplifiers were adjusted to ensure that the negative cell population (*i.e.* non-fluorescent population) appeared in the first two logarithmic decades. An electronic marker was then placed at the limit of the negative control to express all positive cell populations in the final two logarithmic decades. Compensation for spectral overlap between each of the three different fluorescent antibodies used was performed using the protocol stated above for all test samples (with the exception that only 5’000 gated events were acquired for each compensation control) and then electronically calculated via BD FACS Diva (Version 6.0, BD Biosciences, Basel, Switzerland) computational software prior to collecting all data. Sample data was analysed using the gating strategy as shown in SI Fig. 1a and b respectively. It is important to note that this gating strategy considers both the specific characteristics of the side- and forward-scatter (*i.e.* cell size and granularity) of the cell population of interest as well as the fluorescent signature of the sample. All data was analysed using FlowJo (Version 10, TreeStar, USA). Results are presented as the % Frequency. This assay was repeated on three separate occasions in triplicate (n = 3).

Importantly, to further investigate the impact of Trypsin-EDTA upon the phenotype of both immune cell types (MDM and MDDC), control experiments were conducted to confirm the presence and frequency of the specific cell surface proteins used. MDM and MDDC were cultured either with or without EDTA-Trypsin and either as monocultures or within the triple cell co-culture itself. The negative control for all sample analysis was either an unstained cell monoculture (MDM or MDDC) or co-culture suspension having been treated with EDTA-Trypsin or not. For both monocultures and co-cultures, preparation and immunostaining of the cell suspensions was the same as described above. Samples were assessed *via* one-colour FACS for the expression of CD11a and CD14 (MDM) or CD1c and CD80 (MDDC) respectively. Samples were analysed using two-colour FACS (LSR Fortessa (3 laser, 4-blue-2-red-2-violet (405 nm (violet); 488 nm (blue); 640 nm (red)) BD Biosciences, Basel, Switzerland)). Fluorescent signals were collected in logarithmic mode (4 decade logarithmic amplifier) and cell numbers per channel in linear mode. To identify each cell population, an electronic gate was placed around the side- and forward-scatter modes with 30’000 gated events acquired for each sample. The fluorescent amplifiers were adjusted to ensure that the negative cell population (*i.e.* non-fluorescent population) appeared in the first two logarithmic decades. An electronic marker was then placed at the limit of the negative control to express all positive cell populations in the final two logarithmic decades. No compensation was performed for spectral overlap as only one fluorophore (one-colour FACS) was used. Sample data was analysed using the gating strategy as shown in SI Fig. 1a and b respectively. It is important to note that this gating strategy considers both the specific characteristics of the side- and forward-scatter (*i.e.* cell size and granularity) of the cell population of interest as well as the fluorescent signature of the sample. All data was analysed using FlowJo (Version 10, TreeStar, USA). Results are presented as the %Frequency. This assay was repeated on three separate occasions in triplicate (n = 3).

#### Annexin V Assay via FACS

To confirm that the method employed to obtain a cell suspension of the multi-cellular *in vitro* system of the human lung epithelial tissue barrier did not induce any unintended cell death (*e.g.* apoptosis or necrosis), the model system was exposed to *complete medium* for 24 hours at 37 °C, 5% CO_2_. As a positive control for apoptosis, the co-culture was exposed to Camptothecin at [0.002 mg/mL] for 24 hours. Camptothecin (was used due to its known apoptotic nature towards mammalian cells, even at low concentrations^29^). Following the exposure period, cell suspensions were prepared as previously described above. At this point, the positive control for dead cells (*i.e.* necrotic cells) was prepared by placing a suspension sample at −80 °C for 30 minutes (dead cells (*i.e.* necrosis)) as previously described by Blank *et al*.^30^. The negative control for all sample analysis was an unstained co-culture suspension exposed to *complete medium* only. All samples were then washed once with PBS *via* centrifugation (0.5 g for 5 minutes at 4 °C) and then re-suspended in 400 µL FACS buffer. Cell suspensions were then stained by using the Annexin-V-Fluos kit (Roche Diagnostics, Switzerland). Briefly, the co-culture suspensions were stained with equal volumes of Annexin V (apoptosis) and propidium iodide (PI) for dead cells (*i.e.* necrotic cells) and then prepared for two-colour FACS analysis according to the manufacturer’s guidelines. Samples were analysed using two-colour FACS (LSR Fortessa (3 laser, 4-blue-2-red-2-violet (405 nm (violet); 488 nm (blue); 640 nm (red)) BD Biosciences, Basel, Switzerland)). Fluorescent signals were collected in logarithmic mode (4 decade logarithmic amplifier) and cell numbers per channel in linear mode. To identify each cell population, an electronic gate was placed around the side- and forward-scatter modes with 30’000 gated events acquired for each sample. The fluorescent amplifiers were adjusted to ensure that the negative cell population (*i.e.* non-fluorescent population) appeared in the first two logarithmic decades. An electronic marker was then placed at the limit of the negative control to express all positive cell populations in the final two logarithmic decades. Compensation for spectral overlap between each of the three different fluorescent antibodies used was performed using the protocol stated above for all test samples (with the exception that only 5’000 gated events were acquired for each compensation control) and then electronically calculated via BD FACS Diva (Version 6.0, BD Biosciences, Basel, Switzerland) computational software prior to collecting all data. All data was subsequently analysed using FlowJo (Version 10, TreeStar, USA). A representative gating strategy, as described in SI Fig. 1a and b respectively was implemented. It is important to note that this gating strategy considers both the specific characteristics of the side- and forward-scatter (*i.e.* cell size and granularity) of the cell population of interest. Briefly, the results of the total cell suspension analysis were expressed in quadrants categorized according to the Annexin V and PI expressions as healthy (Annexin V/PI −/−), early apoptotic (Annexin V/PI +/−), late apoptotic (Annexin V/PI +/+) and dead (Annexin V/PI −/+). All data was analysed using FlowJo (Version 10, TreeStar, USA). All data is presented as the %Frequency. This assay was repeated on three separate occasions in triplicate (n = 3).

In addition to the two-colour FACS analysis, samples were also subsequently prepared in order to assess the level of cell death in each specific cell type. To achieve this, samples were prepared as stated above, however prior to using the Annexin-V-Fluos kit cell suspension samples were firstly incubated with Fc-block receptor (Miltenyi Biotech (Cat #:130-059901), Switzerland) for 10 minutes in order to inhibit Fc-receptor binding of specific antibodies, then washed x1 with PBS prior to being stained for the specific cell surface proteins CD14 (MDM), Pan-Cytokeratin (A549 epithelial cells) and CD1c (MDDC) and incubated at 4 °C for an additional 30 minutes. For staining specific information please refer to SI Table 1. Following the immunostaining period, samples were washed x1 with PBS *via* centrifugation at 0.5 g for 5 minutes at 4 °C. The cell pellet was then re-suspended in 400 µL FACS buffer. Cell suspensions were then stained with Annexin V and PI, as stated previously and analysed using *via* five-colour FACS (LSR Fortessa (3 laser, 4-blue-2-red-2-violet (405 nm (violet); 488 nm (blue); 640 nm (red)) BD Biosciences, Basel, Switzerland)) using the precise same settings as described for the two-colour FACS Annexin V analysis. Again, the negative control for all sample analysis was either an unstained cell monoculture (MDM, MDDC or A549 epithelial cells) or co-culture suspension. Sample data was analysed using the gating strategy as shown in SI Fig. 1b. Briefly, the results were expressed in quadrants categorized according to the Annexin V and PI expressions against the specific cell population as healthy (Annexin V/PI −/−), early apoptotic (Annexin V/PI +/−), late apoptotic (Annexin V/PI +/+) and dead (Annexin V/PI −/+). Specific compensation was performed for spectral overlap for each fluorophore used, as previously described. All data was analysed using FlowJo (Version 10, TreeStar, USA). Results are also presented the %Frequency. This assay was repeated on three separate occasions in triplicate (n = 3).

### Cell Specific Biochemical Response

#### DCFH-DA Assay via FACS

To deduce the ability for the described technique to elucidate understanding of the cell specific biochemical response, a model toxicology assay was used. Since the onset of oxidative stress, *via* the production of reactive oxygen species (ROS) is a fundamental principle of toxicological research^19^, the formation of ROS was assessed within the co-culture using the fluorescent probe 2′,7′-dichlorfluorecein-diacetate (DCFH_2_-DA) (*i.e.* the DCFH-DA assay)^20^. The DCFH-DA assay is a commonly used method to detect ROS formation within cell and cell-free systems^21^. Principally, the method is based upon the hydrophobic molecule DCFH_2_-DA which can readily diffuse through the cellular membrane and once it interacts with intracellular esterases is hydrolysed and then oxidised to form dichlorofluorescin (DCF), which appears as a strongly fluorescent signal on the FITC channel (488 nm) and is proportional to the amount of ROS formed inside of the cell^31^.

Therefore, to achieve this desired understanding and the applicability of this method towards such biochemical effects, the co-culture, when cultured upon the micro-porous insert, was exposed to either *complete medium* (negative control) or *tert*-butyl hydrogen peroxide (*t*BHP) at [0.04 mg/mL] (positive control) for 4 hours at 37 °C, 5% CO_2_. The concentration used for *t*BHP, a known oxidant able to initiate ROS production^32^, was based on the findings of Clift *et al.* ^11^ who reported significant levels of oxidative stress at this concentration without any cytotoxic effects to the triple cell co-culture. Following the exposure period, samples were then treated with the DCFH-DA fluorescent probe based on the method previously described by Clift *et al.* ^33^. Briefly, cells were washed once with PBS at room temperature and then stained with 2′,7′-dichlorfluorecein-diacetate (488 nm) (diluted 1:4 in methanol to 1 mM and then further diluted 1:10 in PBS) for 30 minutes at 37 °C, 5% CO_2_. A DCFH-DA treated control (DCFH-ve) was also performed to denote any non-specific fluorescence within the sample. Samples were then washed x1 with PBS as before, and co-culture suspensions were obtained *via* the EDTA-Trypsin method as described above. Samples were then either analysed as a total cellular ROS response, or for the cell specific ROS response. To achieve the cell specific response analysis, cells were first treated with Fc-block receptor (Miltenyi Biotech (Cat #:130-059901), Switzerland) for 10 minutes in order to inhibit Fc-receptor binding of specific antibodies, then after x1 wash with PBS were stained for each specific cell type, *i.e.* MDM (CD14), A549 epithelial cells (Pan-Cytokeratin) and MDDC (CD1c) in FACS buffer for 30 minutes at 4 °C. For staining specific information please refer to SI Table 1. Following the staining period, samples were washed x1 with PBS *via* centrifugation at 0.5 g for 5 minutes and the cell pellet then re-suspended in 400 µL FACS buffer. Samples were then analysed using either one- or four-colour FACS (LSR Fortessa (3 laser, 4-blue-2-red-2-violet (405 nm (violet); 488 nm (blue); 640 nm (red)) BD Biosciences, Basel, Switzerland)). Fluorescent signals were collected in logarithmic mode (4 decade logarithmic amplifier) and cell numbers per channel in linear mode. To identify each cell population, an electronic gate was placed around the side- and forward-scatter modes with 30’000 gated events acquired for each sample. The fluorescent amplifiers were adjusted to ensure that the negative cell population (*i.e.* non-fluorescent population) appeared in the first two logarithmic decades. An electronic marker was then placed at the limit of the negative control to express all positive cell populations in the final two logarithmic decades. Compensation for spectral overlap between each of the three different fluorescent antibodies used was performed using the protocol stated above for all test samples (with the exception that only 5’000 gated events were acquired for each compensation control) and then electronically calculated via BD FACS Diva (Version 6.0, BD Biosciences, Basel, Switzerland) computational software prior to collecting all data. All data was subsequently analysed using FlowJo (Version 10, TreeStar, USA). A representative gating strategy, as described in SI Fig. 1a and b respectively was implemented. It is important to note that this gating strategy considers both the specific characteristics of the side- and forward-scatter (*i.e.* cell size and granularity) of the cell population of interest. Briefly, the results were expressed in quadrants and cell populations categorized according to the DCFH expression alone (one-colour work), or against the specific cell population (four-colour work) as no ROS production (DCFH −/−), non-cell associated ROS production (DCFH +/−), cell specific staining (DCFH −/+) and specific cell type associated ROS production (DCFH +/+). All data was analysed using FlowJo (Version 10, TreeStar, USA). All data is presented as the %Frequency. This assay was repeated on three separate occasions in triplicate (n = 3).

Subsequent investigation was performed to order to compare DCFH FACS-based analysis against that obtained *via* the LSM-based method (please refer to next section for details). To achieve this, the cell specific response calculated as the %Frequency from the four-colour FACS analysis (as shown in Fig. 6) for the ROS response was calculated against the respective %Frequency of the negative control (*complete medium* only) and then averaged across the replicates (n = 3) in order to deduce a specific cell type based response at the single cell level relative to the ROS response observed within the co-culture suspension. This can be expressed *via* the following equation:

$${\bf{S}}{{\bf{C}}}_{{\rm{ROS}}}= \% {\bf{f}}/{{\bf{C}}}^{{\rm{pop}}}$$

where **SC**_ROS_ represents the cell specific ROS response, %**f** is the %Frequency determined from the specific cell population of interest (**C** ^pop^).

#### DCFH-DA Assay via Confocal Laser Scanning Microscopy

To compare the response observed using the FACS-based protocol, and further support the notion of the herein presented method, the ability for ROS production to be measured in the co-culture system and within each specific cell type was repeated using confocal laser scanning microscopy (LSM). Specifically live LSM was used in order to compare directly to the live cell cultures analysed *via* FACS. To achieve this, both immune cells were pre-labelled with a fluorescent dye prior to being cultured with the A549 epithelial layer. MDDC were cultured *via* the scrapping method, as previously described above, and then initially stained with a specific live dye (1:100 dilution in PBS; DiD (644/665 nm), Vybrant Multicolor Cell-Labeling Kit (Cat #: V22889), Molecular Probes, Switzerland) for 15 minutes at 37 °C, 5% CO_2_. Following the immunofluorescent staining period, cells were then cultured on the basolateral side of the epithelial layer as described for the co-culture above. After 45 minutes incubation at 37 °C, 5% CO_2_, to allow for effective attachment of the MDDC to the basolateral side of the insert membrane, the MDM were stained with another cell specific live cell dye (1:100 dilution in PBS; DiL (549/565 nm), Vybrant Multicolor, Cell-Labeling Kit (Cat #: V22889), Molecular Probes, Switzerland), again for 15 minutes at 37 °C, 5% CO_2_. MDM were then subsequently cultured on the apical layer of the co-culture and incubated for a further 45 minutes at 37 °C, 5% CO_2_. Co-culture samples were then either treated with (i) *complete* medium (negative control) or (ii) *t*BHP (positive control) at [0.04 mg/mL] for 4 hours at 37 °C, 5% CO_2_. After the exposure period, samples were then either exposed to (i) *complete medium* only (negative control), (ii) DCFH –ve or (iii) DCFH-DA +ve (488 nm) ([1 mM]) solutions for 30 minutes at 37 °C, 5% CO_2_, as previously described for the FACS analysis. Samples were then stained with a nuclear stain mask at 1:10’000 dilution in PBS (Cell Tracker Violet (516 nm) (Cat #: C10094), Molecular Probes, Switzerland) for 45 minutes at 37 °C, 5% CO_2_. Following this final staining period, samples were then washed x2 with PBS and then mounted directly onto microscope slides and covered with a cover-slip only. It is important to note that the cover-slips were not fixed to the microscope slides with any substance as this would have affected the live cell cultures on the membrane inserts. Images were then collected using an inverted LSM 710 Meta (Carl Zeiss, Germany) using a Plan-Apochromat 63x/1.4 lens (NA = 1.3) with 0.3 µm z-stacks to enable the spatial investigation in 3D. Images are shown with the XY plane in a 3D rendered format (as computed using IMARIS®, Switzerland), as well as planar views of XZ shown in 2D in order to confirm the presence, or absence of cells (*i.e.* cellular layer) attached to the membrane insert. The ROS signal identified *via* live LSM was subsequently quantified on a single cell level. Quantification was performed in a two-step procedure. Firstly, the DIL, DiD or Vybrant Multicolor signals were used to create a 3D masked outline of each MDM, MDDC or A549 epithelial cell, respectively. Secondly, each binarized mask was overlayed with the DCF channel of the corresponding cell, which hereby identified the cell type and removed all extracellular fluorescent background. Hence, only pixels inside cell boundaries contribute to the mean pixel intensity of each specific cell type. All image processing was performed using ImageJ^34^. This protocol was repeated on three separate occasions in triplicate (n = 3). At least six cells of each cell type were analysed during each experiment, contributing at least 200 000 pixels to each cell type.

#### Data and Statistical Analysis

All data is presented as the mean ± standard error of the mean (SEM). All experimentation was repeated three times from three independent immune cell isolations (n = 3). Where appropriate, statistical significance was determined upon normally distributed data sets (data not shown) *via* a Student’s *t-*test compared to the negative control (*i.e.* 3D triple cell co-culture or MDM/MDDC/A549 cell monocultures treated with *complete medium* only) (SPSS, IBM, USA). Data sets were considered significant if *p* < 0.05.

## Electronic supplementary material

SI Figures
