# Supplementary material for: A novel technique to determine the cell type specific response within an in vitro co-culture model via multi-colour flow cytometry
Source: Sci Rep. 2017 Mar 27;7:434. doi: 10.1038/s41598-017-00369-4 (PMC5428288; doi:10.1038/s41598-017-00369-4)
Supplement: Supplementary file 1 — SI Figures [file 41598_2017_369_MOESM1_ESM.pdf]

Supplementary Information

Title:

A novel technique to determine the cell type specific response within an *in vitro* co-culture model *via* multi-colour flow cytometry

Authors:

Martin J. D. Clift[*†1,2], Kleanthis Fytianos[†1], Dimitri Vanhecke[1], Sandra Hočevar[1], Alke Petri-Fink[1,3] and Barbara Rothen-Rutishauser[1]

[†] **Authors contributed equally.**

Affiliations:

1. BioNanomaterials, Adolphe Merkle Institute, University of Fribourg, Switzerland.

2. *In Vitro* Toxicology Group, Swansea University Medical School, Wales, United Kingdom

3. Department of Chemistry, University of Fribourg, Switzerland

***Corresponding Author:**

Dr. Martin J. D. Clift, *In Vitro* Toxicology Group, Institute of Life Sciences, Swansea University Medical School, Singleton Park Campus, Swansea SA2 8PP, Wales, United Kingdom; Tel #: 0044 (0)1792 602742; E-mail: m.j.d.clift@swansea.ac.uk

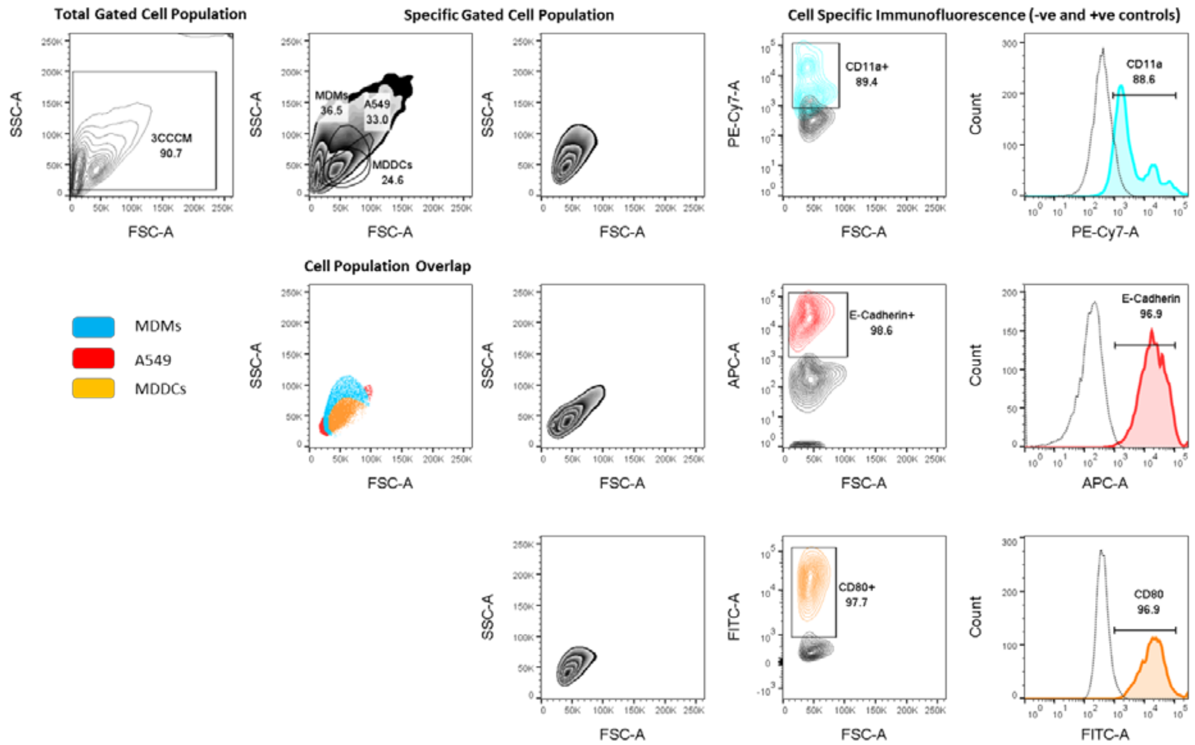

Supplementary Figure 1a: Gating strategy used to determine the specific cell population of each monoculture cell type (*i.e.* MDM, A549 cells and MDDC) analysed (*i.e.* gated cell population). It is important to note that this gating strategy considers both the specific characteristics of the side- and forward-scatter (*i.e.* cell size and granularity) of the cell population of interest as well as the fluorescent signature of the sample. Also shown is the measured fluorescence of each cell type either as the negative control (*complete medium only* (*i.e.* unstained sample)) or cells stained with antibodies for the specific cell surface proteins detailed in SI Table 1; MDM (CD14 and CD11a), A549 cells (Pan-Cytokeratin and E-Cadherin), MDDC (CD1c and CD80). Both the unstained and stained samples are further shown as an overlay (*i.e.* combined) to emphasize the positive fluorescence observed with each of the antibodies used for the specific cell surface proteins of every cell type. All analysis shown was performed using FlowJo (Version 10, TreeStar, USA). The numbers shown in the 'gated cell population' scatter-plots are the percentage of the total acquired cell population (*i.e.* stopping gate) from the sample analysis undertaken using BD FACS Diva (Version 6.0, BD Biosciences, Basel, Switzerland). The numbers presented in the 'unstained sample', 'stained sample' and 'combined' histograms refer to the percentage fluorescence of the population gated (*i.e.* 'gated cell population'). Data shown is of monocultures treated with *complete medium* only at 37°C, 5% CO_2. Data expressed is the mean ± standard error of the mean (SEM) of the %Frequency. Experimentation was repeated on three separate occasions in triplicate (n=3).

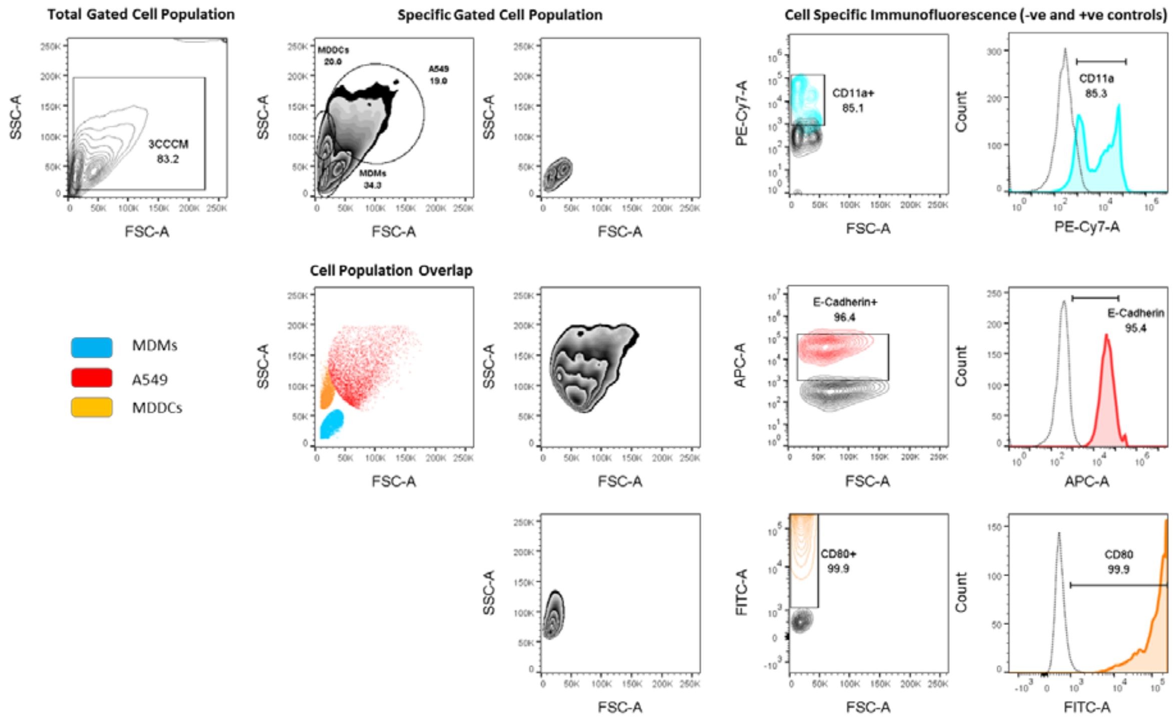

Supplementary Figure 1b: Gating strategy implemented to determine the specific cell population of each cell type (*i.e.* MDM, A549 cells and MDDC) analysed (*i.e.* gated cell population) in the suspension of the 3D *in vitro* triple cell co-culture model of the human epithelial airway barrier. The gating strategy was founded upon that described for monocultures in SI Fig. 1a. It is important to note that this gating strategy considers both the specific characteristics of the side- and forward-scatter (*i.e.* cell size and granularity) of the cell population of interest as well as the fluorescent signature of the sample. The figure shows the use of an initial, general gate to determine a positive population for the total cell population. Within this gate, each cell type a cell specific gate was applied (which was supported through the use of 'back-gating' to confirm the positive fluorescence within the sample cell population). Through this approach, it was possible to specifically compare each cell type against one another in the co-culture model. Also shown is the measured fluorescence of each cell type either as the negative control (*complete medium* only (*i.e.* unstained sample)) or cells stained with antibodies for the specific cell surface proteins detailed in SI Table 1; MDM (CD14 and CD11a), A549 cells (Pan-Cytokeratin and E-Cadherin), MDDC (CD1c and CD80). Both the unstained and stained samples are further shown as an overlay (*i.e.* combined) to emphasize the positive fluorescence observed with the antibodies used for the specific cell surface proteins of each cell type. All analysis shown was performed using FlowJo (Version 10, TreeStar, USA). The numbers shown in the 'gated cell population' scatter-plots are the percentage of the total acquired cell population (*i.e.* stopping gate) from the sample analysis undertaken using BD FACS Diva (Version 6.0, BD Biosciences, Basel, Switzerland). The numbers presented in the 'unstained sample', 'stained sample' and 'combined' histograms refer to the percentage fluorescence of the population gated (*i.e.* 'gated cell population'). All data presented is representative of all samples investigated (n=3). Data shown is of the TCCC treated with *complete medium* only at 37°C, 5% CO_2. Data expressed is the mean ± standard error of the mean (SEM) of the %Frequency. Experimentation was repeated on three separate occasions in triplicate (n=3).

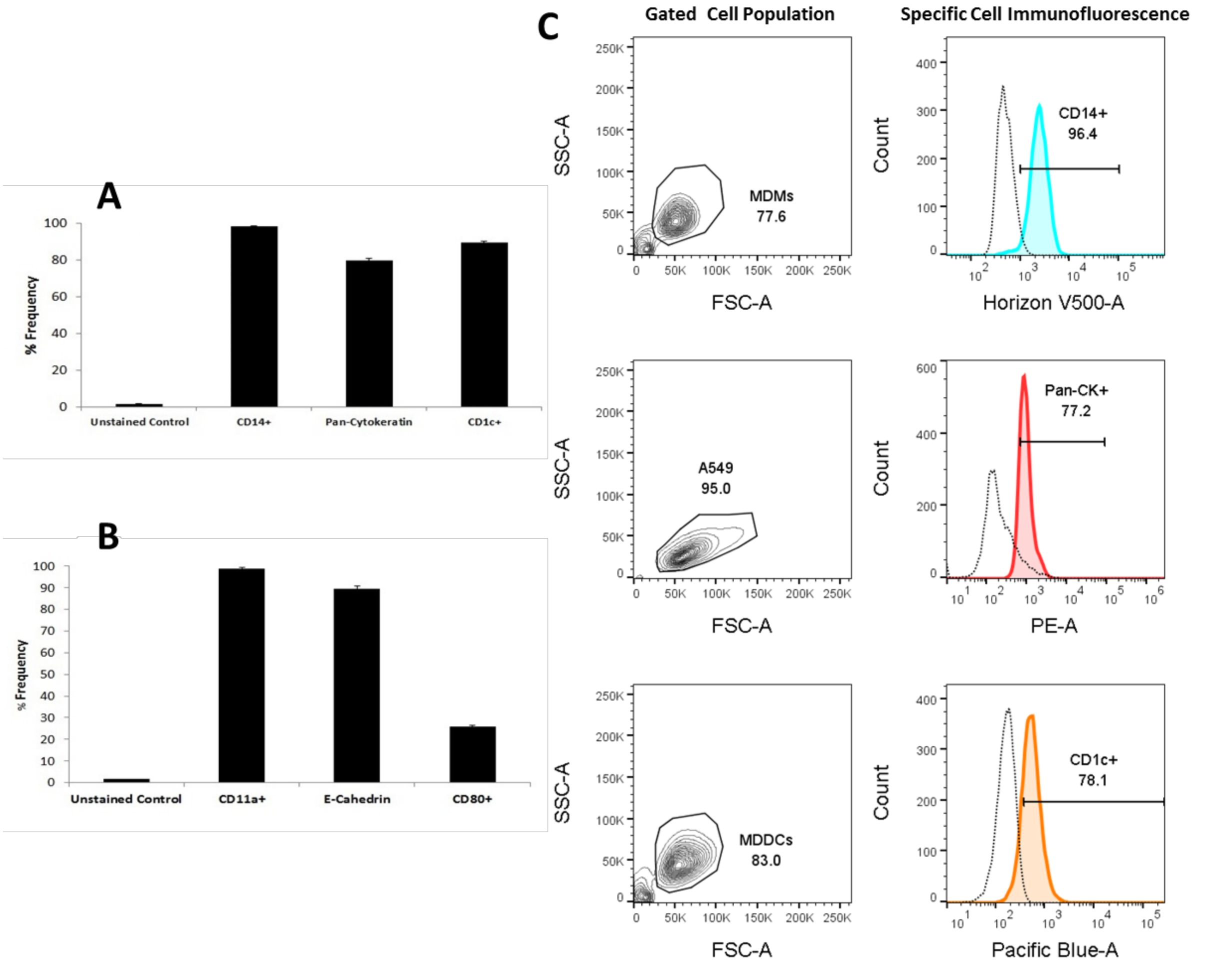

Supplementary Figure 2: Percentage frequency (%Frequency) of the specific surface marker expression on human blood monocyte derived macrophages (MDM) and dendritic cells (MDDC) as well as A549 epithelial cells The specific surface protein markers used are detailed in SI Table 1, however briefly, MDM were stained with either CD14 or CD11a. Epithelial cells were stained for either Pan-Cytokeratin or E-Cadherin, whilst MDDC were identified with either CD1c or CD80. In each graph, the expression of each surface marker is also given in regards to an unstained sample of the same cell type. (B) Shows the expression of the combined staining of the co-culture suspension with CD14 (MDM), Pan-Cytokeratin (A549 cells) and CD1c (MDDC). (**B**) Shows the expression of the surface markers CD11a (MDM), E-Cadherin (A549 cells) and CD80 (MDDC). (**C**) Shows an example gating strategy to define the %Frequency expression of all surface markers analysed. All data was analysed using FlowJo (Version 10, TreeStar, USA) using the gating strategy described for monocultures in SI Fig. 1a (**C**). Data expressed is the mean ± standard error of the mean (SEM) of the %Frequency. Experimentation was repeated on three separate occasions in triplicate (n=3).

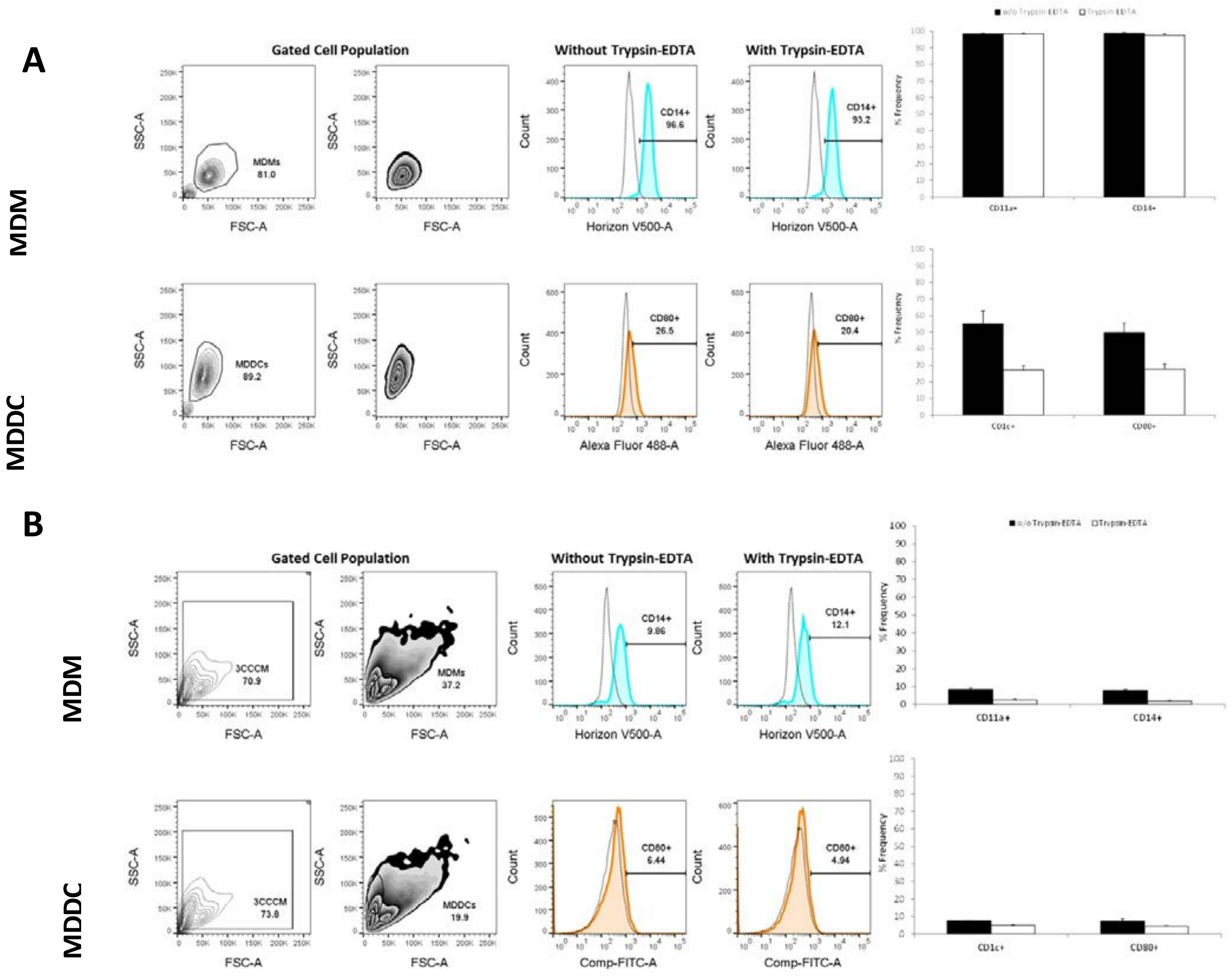

Supplementary Figure 3: Percentage frequency (%Frequency) of the specific surface marker expression on human blood monocyte derived macrophages (MDM) and dendritic cells (MDDC) following either Trypsin-EDTA or no Trypsin-EDTA treatment in (**A**) monocultures or (**B**) the 3D triple cell co-culture. Cells are stained for CD14/CD11a (MDM), or CD1c/CD80 (MDDC). In each graph, the expression of each surface marker is also given in regards to an unstained sample of the same cell type. All data was analysed using FlowJo (Version 10, TreeStar, USA) using the gating strategy described for monocultures in SI Fig. 1a. Data expressed is the mean ± standard error of the mean (SEM) of the %Frequency. Experimentation was repeated on three separate occasions in triplicate (n=3).

SUPPLEMENTARY TABLE 1

SI Table 1: Specific characteristics of the different fluorescent cell surface protein markers used to differentiate each cell type, either as a monoculture or as part of the multi-cell suspension *via* (multi-colour) flow cytometry

(FACS) in the present study. It is important to note that each marker presented is only an example of the fluorophore(s) that can be used to identify each cell type that consist within the *in vitro* triple cell co-culture system of the epithelial airway barrier. Both panels were chosen specifically to fit the filter set available on the FACS used (LSR Fortessa BD Biosciences, Basel, Switzerland) as well as to complement the other fluorescent markers used within the study (*i.e.* within both Annexin V and DCFH-DA assays).

**Specific Cell Type Identified**	**Specific Cell Surface Marker**	**Characteristic of Cell Surface Marker**	**Filter used (BD LSR Fortessa)**	**Working Dilution (FACS buffer*)**	**Supplier**
MDM	Anti-human CD14	Cell Phenotype	Brilliant Violet	50x	BioLegend, USA
MDM	Anti-human CD11a	Immune Cell Co-Stimulatory Marker	PE-Cy7	50x	BioLegend, USA
Epithelial Cells (A549)	Anti-human Pan-Cytokeratin	Associated with Cell Differentiation	PE	10x	Santa Cruz Biotechnology, USA
Epithelial Cells (A549)	Anti-human CD324 (E-cadherin)	Intracellular Adhesion Molecule	FITC	50x	BioLegend, USA
MDDC	Anti-human CD1c	Associated with Immune Cell Activation	Pacific Blue	100x	BioLegend, USA
MDDC	Anti-human CD80	Associated with Immune Cell Activation	PerCP/Cy5.5	100x	BioLegend, USA
